# Supplementary material for: Combined Effects of Carotenoids and Polyphenols in Balancing the Response of Skin Cells to UV Irradiation
Source: Molecules. 2021 Mar 30;26(7):1931. doi: 10.3390/molecules26071931 (PMC8036680; doi:10.3390/molecules26071931)
Supplement: Supplementary file 1 [file molecules-26-01931-s001.pdf]

Table S1. Individual results of plasma lycopene concentrations. Results are in μM.

Treatment		Lycoderm™		Washout	
**Volunteer**	**Baseline**	**Week 2**	**Week 3**	**Week 6**	**Week 7**
1	1.02	2.36	1.69	1.15	1.12
2	0.69	1.56	1.71	1.10	0.95
3	0.54	0.70	0.74	0.49	0.38
4	1.28	2.59	2.99	1.48	1.38
5	1.11	2.57	2.65	1.32	1.08
6	0.83	0.67	1.38	1.70	1.15
7	2.04	1.50	2.11	1.39	0.98
8	1.47	1.78	2.61	1.47	1.38
9	0.66	0.87	1.58		0.81
10	1.31	1.38	1.48	0.98	0.76
11	1.77	1.16	1.84	1.42	1.09
12	0.98	1.72	2.15	1.55	1.19
13	0.57	1.19	1.00	1.18	0.70
14	0.68	1.12		0.68	0.63
15	1.12	1.19	2.06	1.25	0.76
16	0.71	1.31	1.59		1.06
17	1.10	1.51	2.07	1.10	0.72
18	0.87	1.88	1.98	0.82	0.55
19	0.83	2.03	2.17	1.18	0.96
20	0.53	1.27	1.30	1.08	1.24
21	1.13	2.59	3.25	1.50	1.02
22	0.90	1.55	1.79	0.83	0.89
23	1.70	1.77	2.46	1.38	1.38

Table S2. Individual results of plasma phytofluene concentrations. Results are in µM.

Treatment		Lycoderm™		Washout	
**Volunteer**	**Baseline**	**Week 2**	**Week 3**	**Week 6**	**Week 7**
1	0.21	0.59	0.61	0.33	0.31
2	0.15	0.46	0.55	0.36	0.26
3	0.15	0.23	0.30	0.17	0.15
4	0.26	0.52	0.70	0.34	0.39
5	0.34	1.11	1.28	0.59	0.39
6	0.29	0.25	0.34	0.50	0.34
7	0.43	0.39	0.66	0.35	0.27
8	0.23	0.25	0.40	0.19	0.19
9	0.20	0.36	0.65		0.23
10	0.19	0.34	0.52	0.29	0.19
11	0.48	0.46	0.90	0.40	0.32
12	0.16	0.34	0.56	0.39	0.23
13	0.18	0.43	0.22	0.42	0.27
14	0.08	0.14		0.09	0.09
15	0.28	0.23	0.51	0.26	0.17
16	0.21	0.51	0.27		0.45
17	0.19	0.33	0.62	0.23	0.18
18	0.22	0.29	0.50	0.30	0.21
19	0.14	0.30	0.32	0.21	0.18
20	0.18	0.46	0.46	0.24	0.29
21	0.20	0.77	1.00	0.34	0.20
22	0.10	0.30	0.41	0.13	0.20
23	0.74	0.57	1.37	0.39	0.44

Table S3. Individual results of plasma phytoene concentrations. Results are in μM.

Treatment		Lycoderm™		Washout	
**Volunteer**	**Baseline**	**Week 2**	**Week 3**	**Week 6**	**Week 7**
1	0.04	0.20	0.20	0.04	0.06
2	0.02	0.12	0.16	0.03	0.04
3	0.02	0.10	0.10	0.02	0.03
4	0.06	0.19	0.28	0.07	0.06
5	0.07	0.45	0.37	0.09	0.05
6	0.09	0.07	0.10	0.14	0.06
7	0.11	0.12	0.16	0.10	0.05
8	0.06	0.10	0.16	0.04	0.03
9	0.02	0.09	0.22		0.04
10	0.08	0.21	0.20	0.06	0.04
11	0.06	0.13	0.21	0.04	0.05
12	0.02	0.15	0.17	0.05	0.03
13	0.02	0.12	0.10	0.06	0.03
14	0.02	0.06		0.01	0.02
15	0.06	0.09	0.21	0.05	0.02
16	0.06	0.21	0.19		0.16
17	0.05	0.15	0.36	0.04	0.03
18	0.07	0.19	0.30	0.08	0.05
19	0.04	0.12	0.10	0.04	0.04
20	0.03	0.22	0.18	0.03	0.05
21	0.04	0.28	0.33	0.04	0.03
22	0.03	0.13	0.19	0.01	0.03
23	0.19	0.27	0.45	0.15	0.13

Table S4. Individual results of plasma β-carotene concentrations. Results are in μM.

Treatment		Lycoderm™		Washout	
**Volunteer**	**Baseline**	**Week 2**	**Week 3**	**Week 6**	**Week 7**
1	0.65	1.03	0.83	1.05	1.22
2	0.96	1.48	1.18	1.28	0.97
3	0.66	0.50	0.43	0.66	0.56
4	0.56	0.60	0.68	0.84	0.62
5	0.82	1.06	0.99	1.40	0.99
6	1.68	0.89	1.38	1.85	1.67
7	1.36	1.29	1.64	1.68	0.89
8	0.83	0.62	0.79	0.72	0.56
9	1.13	0.85	1.20		1.12
10	0.72	0.70	0.79	0.59	0.50
11	1.70	1.32	1.87	2.11	1.55
12	1.08	1.04	1.45	1.83	1.25
13	0.50	0.57	0.52	0.61	0.47
14	0.37	0.44		0.32	0.33
15	0.92	0.62	1.05	1.16	0.91
16	0.63	0.54	0.55		0.89
17	0.48	0.53	0.66	0.45	0.34
18	1.47	1.26	1.66	1.79	1.29
19	0.51	0.66	0.75	0.47	0.48
20	0.52	0.67	0.66	0.53	0.51
21	0.53	0.72	1.01	0.57	0.51
22	0.31	0.40	0.53	0.36	0.36
23	1.43	1.35	1.69	1.17	1.14
